# Cerebral sinovenous thrombosis as a complication of otitis media

**DOI:** 10.1002/ccr3.1948

**Published:** 2018-12-04

**Authors:** Shafee Salloum, Kira Belzer

**Affiliations:** ^1^ Department of Pediatric Hospital Medicine Dayton Children's Hospital Dayton Ohio; ^2^ Pediatric resident PGY‐2 Dayton Children's Hospital Dayton Ohio

**Keywords:** cerebral venous thrombosis, intracranial pressure, mastoiditis, otitis media

## Abstract

Otogenic cerebral sinovenous thrombosis (CSVT) is a rare complication of otitis media and associated with significant morbidity and mortality. Classic clinical signs of mastoiditis (pain, swelling, and erythema posterior to the pinna) are not always present at presentation. Treatment of otogenic CSVT consists of conservative surgery, antibiotics, and anticoagulation.

## 1 CASE PRESENTATION

We report a case of 6‐year‐old girl who presented to our hospital with nonspecific symptoms of fever, headache, and vomiting. She was ultimately diagnosed with cerebral sinovenous thrombosis (CSVT) and was managed medically with antibiotics, anticoagulation, and acetazolamide. Surgical intervention was also required and included lumbar drainage and mastoidectomy with myringotomy.

**Figure 1:**
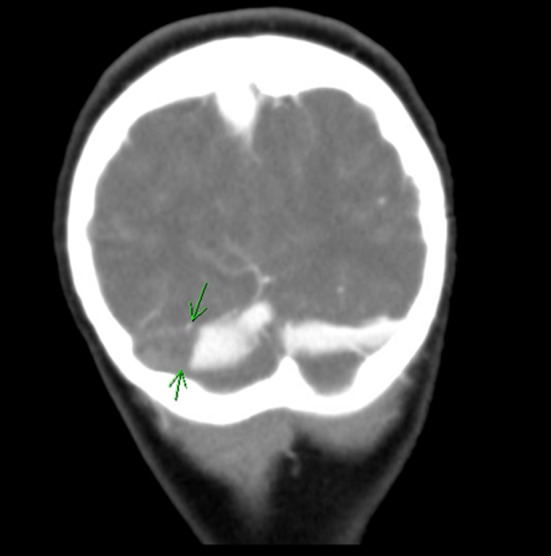
Head CT with contrast arrows indicate the thrombosis within the right transverse sinus; minimal contrast is seen past the thrombosis indicating near‐complete obstruction of the vessel

A 6‐year‐old girl presented to the emergency department with 2 days of fever, vomiting, and headache. She was seen at her pediatrician's office on the day prior to presentation and prescribed amoxicillin for right acute otitis media (AOM). She had bilateral myringotomy tube placement 7 months prior to presentation due to chronic ear infections, otherwise she was healthy and up to date on her immunizations. The patient had no known sick contacts. Family history was significant for maternal history of miscarriages, methylene tetrahydrofolate reductase (MTHFR) mutation, and antiphospholipid antibody syndrome. On arrival; temperature was 36.6°C, heart rate was 70 beats/min, blood pressure was 110/60 mm Hg, respiratory rate was 22 breaths/min with oxygen saturation of 99% on room air. Physical examination revealed a sleepy but easily arousable child. Pupils were round, reactive to light, and extra‐ocular muscles were intact. No nuchal rigidity was noted. Both tympanic membranes were erythematous and bulging, but there was no erythema or tenderness overlying the mastoid processes and no protrusion of the pinna bilaterally. The remainder of her physical examination was unremarkable. The patient was initially treated with intravenous (IV) fluids for dehydration, IV Ketorolac for pain, and ceftriaxone for AOM prior to admission to the pediatric ward. Over the following day, her headache progressed, and she developed diplopia noted when she began covering one eye to watch television. Ophthalmic examination revealed bilateral papilledema consistent with increased intracranial pressure (ICP). Computed tomography (CT) of the head with IV contrast showed CSVT with right mastoiditis Figure 1.

## 2 INVESTIGATION

**Figure 2:**
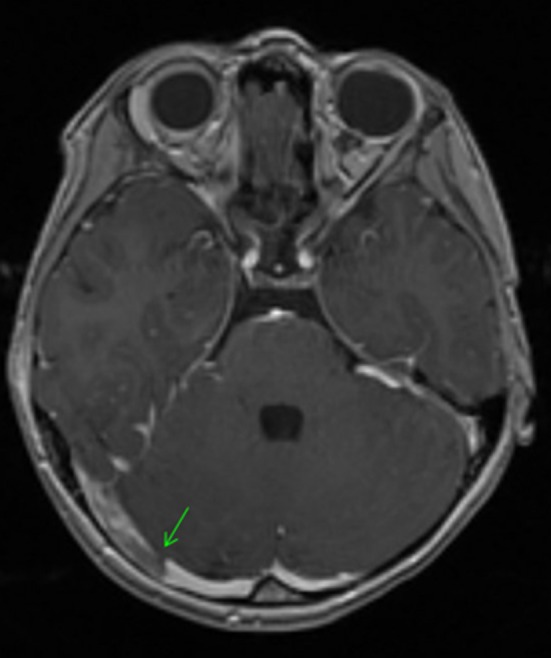
MRI with contrast performed 10 d following initial imaging. Arrow indicates the thrombus within the transverse sinus. Interval decrease in size as indicated by the contrast now visualized beyond the thrombosis

Initial laboratory studies showed low sodium at 133 mmol/L (135‐145), otherwise electrolytes were within normal physiologic parameters. Blood urea nitrogen was elevated at 23 mg/dL (8‐18), but creatinine was normal at 0.4 mg/dL. White blood cell count was normal at 9.6 x 10^3^/mm^3^, but with elevated neutrophils of 80%. Hemoglobin and hematocrit were 13.5 g/dL and 39.4%,respectively, and platelets were normal at 305 × 10^3^/mm^3^. CT of the head with IV contrast revealed right transverse and sigmoid sinuses thrombosis with near‐complete opacification of the right mastoid and middle air cavity Figure 1. Magnetic resonance venography (MRV) with contrast was obtained 10 days later and showed an interval decrease in the right transverse and sigmoid sinuses thrombosis with contralateral hypoplasia of left transverse and sigmoid sinuses Figure 2. Culture of the right ear aspirate grew pseudomonas aeruginosa. A thrombophilia work‐up was obtained and showed normal factor V Leiden, protein C, protein S, prothrombin G20210A, and homocysteine levels. Patient had normal MTHFR A1298C, but she was homozygous for C677T.

## 3 TREATMENT

After diagnosis, the patient was taken to the operating room for right myringotomy with tube placement and mastoidectomy. She underwent therapeutic lumbar puncture that showed an elevated opening pressure of 46 cm H_2_O. She was started on acetazolamide and ultimately required lumbar drainage to alleviate the increased ICP. Low molecular weight heparin (LMWH) was started as well. Antibiotic therapy was changed to cefepime once the ear culture resulted as pseudomonas aeruginosa.

## 4 OUTCOME AND FOLLOW‐UP

Patient was discharged from the hospital 20 days after admission. She had a complete recovery, and at time of discharge, her neurologic and cognitive function had returned to baseline. She completed a total of 6 weeks of IV cefepime, and LMWH was continued for 6 months with interval imaging to assess for resolution of the thrombosis.

## 5 DISCUSSION

Cerebral sinovenous thrombosis is a rare condition in children with a reported incidence of 0.7 per 100,000 children per year.^1^ A high proportion (35%‐43%) of cases occur in newborns due to multiple risk factors in this age‐group including infections, birth trauma, hypoxic injury, dehydration, cardiac defects, and prothrombotic states.^1^, ^2^ Chronic illnesses, such as inflammatory bowel disease, nephrotic syndrome, systemic lupus erythematous, and leukemia (especially when treated with L‐asparaginase), increase the risk of CSVT in older children.^3^, ^4^, ^5^ Acute head and neck infections (otitis media, mastoiditis, and sinusitis) represent the most common predisposing factors in previously healthy children.^2^, ^3^, ^4^, ^5^ A study by Ichord et al,^2^ that studied patients ages 1 month to 19 years, found otitis media, mastoiditis, and sinusitis were risk factors for developing CSVT in 78 of 169 patients (46%). In another study that evaluated 42 children with CSVT, ages 3 weeks to 13 years,Sebire et al^4^ found a recent diagnosis of mastoiditis in 20 of the patients (47%). While many patients develop CSVT secondary to an acute mastoiditis, it remains a rare complication of mastoiditis and patients often have more than one predisposing condition. Anthonsen et al^6^ found no cases of CSVT in 214 children who were admitted for acute mastoiditis, whereas Ghosh et al^7^ found CSVT in 13 of 474 (2.7%) pediatric patients diagnosed with otitis media and mastoiditis. The pathophysiology behind otogenic CSVT is contiguous spread of the inflammatory process from the middle ear, through the mastoids and into the transverse and sigmoid sinuses. Thrombosis of the cerebral sinuses impedes blood flow and causes increased venous pressure which in turn results in increased ICP. In some cases,retrograde venous pressure decreases cerebral blood flow causing cerebral ischemia and infarction.^5^, ^8^, ^9^ Clinical features can be subtle and nonspecific, such as ear pain, fever, headache, irritability, and vomiting. As the condition progresses, signs of increased ICP become more evident such as diplopia (cranial nerve VI palsy), papilledema, altered mental status, seizure, and coma.^3^, ^4^, ^5^ Mastoiditis is not always clinically evident at the time of presentation. In a case series of eight children with otogenic CSVT, Zanoletti et al^9^ reported no clinical signs of acute mastoiditis in three patients (37.5%), which is consistent with our patient's presentation. Prothrombotic condition was reported in 20% of patients with CSVT in a study by Ichord et al,^2^ and in the same study, MTHFR mutation was found in 7% of patients. Wong et al^8^ studied 190 patients with otogenic CSVT,only 50 patients were tested for thrombophilia and 28 of patients tested (56%) were found to have an abnormality. MTHFR mutation was found in five of these patients (10%). Diagnosis of otogenic CSVT requires a high index of suspicion at the beginning as symptoms can be nonspecific as mentioned above. Normal head CT cannot exclude CSVT,even enhanced CT misses the diagnosis of CSVT in up to 40% of cases.^3^, ^4^ The imaging modalities of choice are CT angiography or MRV. Treatment of otogenic CSVT, as we saw in this case, consists of maintaining hydration status, pain control, antibiotics, and measures to alleviate increased ICP such as administering acetazolamide or lumbar drainage.^8^, ^9^ The American Stroke Association recommends LMWH in children with CSVT outside the neonatal period, even in the presence of intracranial hemorrhage.^10^ Surgical intervention is usually conservative and includes mastoidectomy and/or myringotomy.^8^, ^9^ Otogenic CSVT carries a high mortality rate of 5%‐10%^7^ and can lead to significant morbidity and long‐term neurological deficits. In a follow‐up of 106 pediatric patients with CSVT, Wong et al,^8^ reported “symptom free” and “good outcomes” in 78 patients (74%). In 42 patients with CSVT, Sebire et al^4^ found that lateral/sigmoid sinuses involvement, which is usually the case in otogenic CSVT, is associated with better cognitive outcomes. Other factors associated with improved outcomes included older age and no brain parenchymal abnormalities.

## CONFLICT OF INTEREST

The authors have no conflicts of interest to disclose.

## AUTHOR CONTRIBUTION

KB: wrote the case presentation, investigation, and treatment. SS: edited the manuscript and wrote the discussion.

Patient consent: Obtained.
